# Puromycin-based purification of cells with high expression of the cytochrome P450 CYP3A4 gene from a patient with drug-induced liver injury (DILI)

**DOI:** 10.1186/s13287-021-02680-4

**Published:** 2022-01-10

**Authors:** Shoko Miyata, Noriaki Saku, Saeko Akiyama, Palaksha Kanive Javaregowda, Kenta Ite, Nagisa Takashima, Masashi Toyoda, Kei Yura, Tohru Kimura, Hiroshi Nishina, Atsuko Nakazawa, Mureo Kasahara, Hidenori Nonaka, Tohru Kiyono, Akihiro Umezawa

**Affiliations:** 1 Center for Regenerative Medicine, National Center for Child Health and Development Research Institute, Tokyo, 157-8535 Japan; 2 Department of Developmental and Regenerative Biology, Medical Research Institute, Tokyo Medical and Dental University, Tokyo, 113-8510 Japan; 3 Department of Molecular Pathology, Tokyo Medical University, Tokyo, 160-8402 Japan; 4 Research Team for Geriatric Medicine (Vascular Medicine), Tokyo Metropolitan Institute of Gerontology, Tokyo, 173-0015 Japan; 5 Department of BioSciences, Kitasato University School of Science, Kanagawa, 252-0373 Japan; 6 Organ Transplantation Center, National Center for Child Health and Development, Tokyo, 157-8535 Japan; 7 Project for Prevention of HPV-Related Cancer, Exploratory Oncology Research and Clinical Trial Center, National Cancer Center, Chiba, 277-8577 Japan; 8 Advanced Pediatric Medicine, Tohoku University School of Medicine, Miyagi, 980-8574 Japan; 9 Graduate School of Humanities and Sciences, Ochanomizu University, Tokyo, 112-8610 Japan; 10 School of Advanced Science and Engineering, Waseda University, Tokyo, 162-0041 Japan

**Keywords:** Hepatocytes, Cytochrome P450 3A4 (CYP3A4), Drug-metabolizing enzymes, Drug-induced liver injury (DILI), Induced pluripotent stem cells, Immortalization

## Abstract

**Background:**

Many drugs have the potential to induce the expression of drug-metabolizing enzymes, particularly cytochrome P450 3A4 (CYP3A4), in hepatocytes. Hepatocytes can be accurately evaluated for drug-mediated CYP3A4 induction; this is the gold standard for in vitro hepatic toxicology testing. However, the variation from lot to lot is an issue that needs to be addressed. Only a limited number of immortalized hepatocyte cell lines have been reported. In this study, immortalized cells expressing CYP3A4 were generated from a patient with drug-induced liver injury (DILI).

**Methods:**

To generate DILI-derived cells with high expression of CYP3A4, a three-step approach was employed: (1) Differentiation of DILI-induced pluripotent stem cells (DILI-iPSCs); (2) Immortalization of the differentiated cells; (3) Selection of the cells by puromycin. It was hypothesized that cells with high cytochrome P450 gene expression would be able to survive exposure to cytotoxic antibiotics because of their increased drug-metabolizing activity. Puromycin, a cytotoxic antibiotic, was used in this study because of its rapid cytocidal effect at low concentrations.

**Results:**

The hepatocyte-like cells differentiated from DILI-iPSCs were purified by exposure to puromycin. The puromycin-selected cells (HepaSM or SI cells) constitutively expressed the CYP3A4 gene at extremely high levels and exhibited hepatocytic features over time. However, unlike primary hepatocytes, the established cells did not produce bile or accumulate glycogen.

**Conclusions:**

iPSC-derived hepatocyte-like cells with intrinsic drug-metabolizing enzymes can be purified from non-hepatocytes and undifferentiated iPSCs using the cytocidal antibiotic puromycin. The puromycin-selected hepatocyte-like cells exhibited characteristics of hepatocytes after immortalization and may serve as another useful source for in vitro hepatotoxicity testing of low molecular weight drugs.

**Supplementary Information:**

The online version contains supplementary material available at 10.1186/s13287-021-02680-4.

## Background

The toxicity of low molecular weight drugs has been examined in animals such as rats and mice as preclinical safety tests [1]. Primary human cells are a useful tool as an in vitro model for toxicity and are the gold standard in clinical pharmaceutical in vitro studies. However, using human cells in drug screening has its drawbacks, such as a limited supply of any one lot and large variations between lots due to genetic and environmental backgrounds. To solve the issue of lot variation, HepG2 cells are commonly used to examine hepatotoxicity because, they are clonal by nature. HepaRG, another hepatocyte-like clonal cell line, was established from a hepatoblastoma and has an advantage due to its highly inducible cytochrome P450 genes [2]. With the induced pluripotent stem cell (iPSC) technology, hepatocytes can be generated from patients with drug-induced liver injury (DILI), making them a third candidate for experimental cells.

Human iPSCs impact numerous medical fields including clinical therapy development, drug discovery, research on inherited diseases, and studies on reprogramming of differentiated cells [3–6]. For example, iPSC-derived hepatocytes have been shown to serve as an in vitro tool for understanding drug metabolism and toxicology [7–9]. iPSC-derived hepatocytes or hepatocyte-like cells can be obtained from a single origin repeatedly due to the immortality of iPSCs [10–12]. Although it is expected that hepatocytes differentiated from iPSCs can be utilized in drug toxicity testing, the actual applicability of iPSC-derived hepatocytes in this context has not been well examined so far. In this study, iPSCs were generated from pediatric patients with DILI (DILI-iPSCs), and DILI-iPSCs were matured into hepatocytes (DILI-hepatocytes) and other epithelial cells that would be applicable for drug toxicity testing.

Numerous human cells have successfully been immortalized. Human foreskin fibroblasts can be immortalized with telomerase reverse transcriptase (TERT) alone [13]. Human bone marrow stromal cells must be immortalized with TERT and the E6 and E7 genes of human papillomavirus type 16, because telomerase activation by TERT alone is not sufficient to prolong their life span [14, 15]. Human umbilical cord blood-derived mesenchymal stem cells can also be immortalized [16]. In addition to mesenchymal stromal cells and fibroblasts, epithelial cells such as mammary cells, ovarian surface epithelial cells, pancreatic ductal cells, amniotic epithelium, keratinocytes, and hepatocytes have also been immortalized with p16/Rb inactivation by E7 or CDK4/cyclin D and telomerase activation by E6 or TERT [17–20].

Primary human hepatocytes, the gold standard for in vitro testing of clinical drugs, have significant drawbacks such as the limited supply of a single lot and lot-to-lot variability due to genetic and environmental backgrounds. iPSC technology allows the generation of hepatocytes from patients with DILI, making it another source of cells for testing. However, the process of iPSC differentiation into hepatocytes often results in a heterogeneous population of cells including the target cell type. In this study, it was shown that puromycin, a cytocidal antibiotic, can be used to purify hepatocytes that have intrinsic drug-metabolizing enzymes. The purified hepatocyte-like cells exhibit hepatocytic characteristics and may be an in vitro model for the evaluation of hepatotoxicity caused by drug metabolites.

## Methods

### Cells

The differentiated cells from DILI-iPSCs were immortalized by infection with the lentiviral vector CSII-CMV-Tet-Off, CSII-TRE-Tight-cyclin D1, CSII-TRE-Tight-CDK4^R24C^, and CSII-TRE-Tight-TERT [21, 22]. In brief, the EF1α promoter in CSII-EF-RfA (a gift from Dr. H. Miyoshi, RIKEN) was replaced with a tetracycline-inducible promoter, TRE-Tight, from pTRE-Tight (Clontech, Mountain View, CA, USA) to generate CSII-TRE-Tight-RfA. The EF1α promoter in CSII-EF-RfA was replaced with a 2335 bp mouse albumin promoter from pALB-GFP (#55759, Addgene, MA, USA) [23] to generate CSII-ALB-RfA. Human cyclin D1, human mutant CDK4 (CDK4^R24C^: an INK4a-resistant form of CDK4), and TERT were inserted into the entry vector via a BP reaction (Invitrogen, Carlsbad, CA, USA). These segments were then recombined with CSII-TRE-Tight-RfA through an LR reaction (Invitrogen, Carlsbad, CA, USA) to generate CSII-TRE-Tight-cyclin D1, CSII-TRE-Tight-CDK4^R24C^, and CSII-TRE-Tight-TERT. The rtTA segment from pTet-Off Advanced (Clontech, Mountain View, CA, USA) was amplified by PCR, recombined with the donor vector pDONR221 via a BP reaction (Invitrogen, Carlsbad, CA, USA) to generate pENTR221-Tet-Off, and then recombined with a lentiviral vector, CSII-CMV-RfA, and CSII-ALB-RfA, respectively, through an LR reaction (Invitrogen) to generate CSII-CMV-Tet-Off and CSII-ALB-Tet-Off, respectively. Recombinant lentiviruses with vesicular stomatitis virus G glycoprotein were produced as described previously [24]. EpCAM-positive cells were isolated as described below after the immortalization from a mixed population. The immortalized cells were maintained in the modified F-medium at 37 °C in a humidified atmosphere containing 95% air and 5% CO_2_ [25]. When the cultures reached sub-confluence, the cells were harvested with a trypsin–EDTA solution (cat#23315, IBL CO., Ltd, Gunma, Japan), and re-plated at a density of 5 × 10^5^ cells in a 100-mm dish. Medium changes were carried out twice a week thereafter.

### Isolation of EpCAM-positive cells

EpCAM-positive cells were isolated from differentiated endodermal cells using a magnetic cell sorting kit (MACS; Miltenyi Biotec K.K. Cologne, Germany) with the CD326 (EpCAM) MicroBeads (cat# 130-061-101, Miltenyi Biotec, BG, Germany), according to the manufacturer’s instructions. The magnetically labeled EpCAM-positive cells were eluted as a positively selected cell fraction.

### Immunoblot analysis

Whole-cell protein extracts were used for analysis, and immunoblotting was conducted as described previously [22]. Antibodies against E-cadherin (Mouse MAb IgG2a, 610181, BD Transduction Lab, USA), p53 (MAb clone DO-1; IgG2a; OP43-100UG, Calbiochem, Merck KGaA, Darmstadt, Germany), Cyclin D1 (MAb clone G124-326; IgG1; 554180, BD Pharmingen, USA), Vinculin (mouse IgG1; V9264, Sigma-Aldrich, USA), MCM7 (Mouse MAb IgG1; sc-9966, Santa Cruz Biotechnology, USA), CDK4 (Rabbit Pab; Cell Signalling; 610147, BD Transduction Laboratories, USA) were used as probes, and horseradish peroxidase-conjugated anti-mouse or anti-rabbit immunoglobulins (Jackson Immunoresearch Laboratories, PA, USA) were employed as secondary antibodies. The LAS3000 charge-coupled device (CCD) imaging system (Fujifilm Co. Ltd., Tokyo, Japan) was employed for the detection of proteins visualized by Lumi-light Plus Western blotting substrate (Roche, Basel, Switzerland).

### Immunocytochemical analysis

Cells were fixed with 4% paraformaldehyde in PBS for 10 min at 4 °C. After washing with PBS and treatment with 0.2% Triton X in PBS for 10 min, cells were pre-incubated with blocking buffer (10% goat serum in PBS) for 30 min at room temperature and then exposed to primary antibodies to albumin (diluted at 1/50, CLFAG2140, Cedarlane Laboratories, Burlington, Canada), α-fetoprotein (diluted at 1/100, MAB1368, R&D Systems, MN, USA), and CYP3A4 (diluted at 1/200, sc-53850, Santa Cruz Biotechnology, USA) in blocking buffer overnight at 4 °C. Following washing with 0.2% PBST, cells were incubated with secondary antibodies; either anti-rabbit or anti-mouse IgG conjugated with Alexa 488 or 546 (1:300) (Invitrogen) in blocking buffer for 30 min at room temperature. Then, the cells were counterstained with DAPI.

### Karyotypic analysis

Karyotypic analysis was contracted out to Nihon Gene Research Laboratories Inc. (Sendai, Japan). Metaphase spreads were prepared from cells treated with 100 ng/mL of Colcemid (KaryoMax, Gibco, USA) for 6 h. The cells were fixed with methanol/glacial acetic acid (2:5) three times and placed onto glass slides (Nihon Gene Research Laboratories Inc., Miyagi, Japan). Giemsa banding was applied to metaphase chromosomes. A minimum of 10 metaphase spreads was analyzed for each sample and karyotyped using a chromosome imaging analyzer system (Applied Spectral Imaging, Carlsbad, CA, USA).

### Quantitative RT-PCR

RNA was extracted from cells using the ISOGEN (NIPPON GENE, Tokyo, Japan). An aliquot of total RNA was reverse transcribed using an oligo (dT) primer (SuperScript TM III First-Strand Synthesis System, Invitrogen, Merck KGaA, Darmstadt, Germany). For the thermal cycle reactions, the cDNA template was amplified (QuantStudio TM 12 K Flex Real-Time PCR System) with gene-specific primer sets (Additional file 1: Table S1) using the Platinum Quantitative PCR SuperMix-UDG with ROX (11,743–100, Invitrogen, Merck KGaA, Darmstadt, Germany) under the following reaction conditions: 40 cycles of PCR (95 °C for 15 s and 60 °C for 1 min) after an initial denaturation (50 °C for 2 min and 95 °C for 2 min). Fluorescence was monitored during every PCR cycle of the annealing step. The authenticity and size of the PCR products were confirmed using a melting curve analysis (using software provided by Applied Biosystems) and gel analysis. mRNA levels were normalized using ubiquitin as a housekeeping gene. Relative expression levels of each gene are shown. cDNAs from primary hepatocytes were purchased from Takara Shuzo, Ltd (CLN636531 Z6531N, Kyoto, Japan).

### Histological analysis

Cells were harvested with a cell scraper and collected into tubes. The cells were fixed in 4% paraformaldehyde for 10 min at 4 °C. The cells were analyzed with an iPGell kit (GenoStaff, Tokyo, Japan). Paraffin-embedded tissue was sliced and stained with hematoxylin and eosin.

### CYP3A4 induction test

The puromycin-selected immortalized cells were treated with 50 μM omeprazole (solvent: DMSO, 158–03,491, Fujifilm Wako Pure Chemicals, Osaka, Japan) for 24 h, with 500 μM phenobarbital (PB, Wako, Osaka, Japan) for 48 h, or 20 μM rifampicin (RFP, Wako, Osaka, Japan) for 48 h. Controls were treated with DMSO (final concentration 0.2%).

### Enzyme activity measurement

CYP3A4 enzyme activity was measured using the P450-Glo Assay kit (Promega). Luciferin (1 μM) was added to confluent cells for 1 h in a 5% CO_2_ incubator at 37 °C. Then, the medium was transferred to a 96-well white plate, luciferin detection reagent was added, and luminescence intensity was measured with a luminometer.

### Statistical analysis

The numbers of biological and technical replicates are shown in the figure legends. All data are presented as mean ± SD. For most statistical evaluations in this study, an unpaired two-tailed Student's *t*-test was used to calculate statistical probabilities. *P*-values were calculated by a two-tailed *t*-test.

### Microarray analysis

Gene chip analysis was performed as previously described [26]. Total RNA was isolated using a miRNeasy mini kit (217004, Qiagen, Hilden, Germany). RNA samples were labeled and hybridized to a SurePrint G3 Human GEO microarray 8 × 60 K Ver 3.0 (Agilent, CA, USA), and the raw data were normalized using the 75-percentile shift. Gene chip analysis was performed on lymphangioma-derived cells (1: Lym1915-NC, 2: Lym-0 h, 3: Lym-12 h, 4: Lym-24 h, 5: Lym-72 h), pluripotent stem cells (6: SEES5P37 ESC, 7: Edom iPSCs, 8: G72-1 iPSCs, 9: G72-1Shh iPSC, 10: G72-1Vis iPSC, 11: pHAES5-1 ESC) [26–32], HepaSM cells (12: HepaSM), and hepatocytes (13: Saeko_hP3, 14: Saeko_hP10, 15: Ruri_hP4, 16: Ruri_hP10).

### Principal component analysis (PCA)

PCA was performed to determine if puromycin-selected cells derived from iPSCs were in the same cluster as ESC-derived hepatocytes. For the analysis, expression data from 58,201 genes were used. PCA was performed with whole genes using the R statistical package version 3.6.2 (https://www.r-project.org/).

## Results

### Generation of endodermal cells from iPSCs

**Fig. 1:**
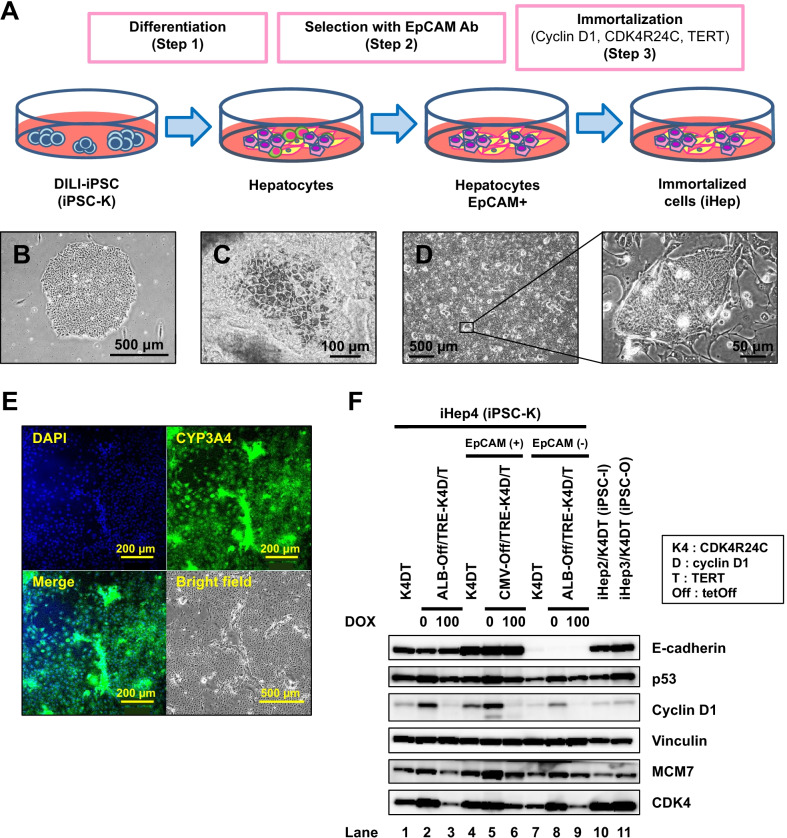
Immortalization of hepatocytes from iPSCs. **A** Scheme for generation of immortalized hepatocytes from iPSCs. Hepatocyte cells are induced from iPSCs (step 1). To immortalize hepatocyte cells, EpCAM-positive cells are sorted from iPSC-derived hepatocytes (step 2), and are infected with the lentiviral vectors carrying TERT, CDK4^R24C^, and cyclin D1 (step 3). **B** Phase-contrast micrograph of iPSC-K. iPSC-K cells on MEFs formed a flat and well-defined colony. **C** Phase-contrast micrograph of hepatocyte-like cells from iPSC-K. **D** Phase-contrast micrograph of immortalized iHep4 cells. **E** Immunocytochemistry of iHep4 cells using an antibody to CYP3A4. **F** Western blot analysis. Samples starting from left: K4DT (iHep4-E4-72 cells infected with CSII-CMV-hCDK4R24C, CSII-CMV-cyclin D1, CSII-CMV-hTERT), (iHep4-E4-74 cells infected with CSII-ALB-tetOff-ADV, CSII-TRE-Tight-cyclin D1, CSII-TRE-Tight-hCDK4R24C, CSII-CMV-hTERT), (iHep4-E8-50 cells infected with CSII-CMV-TetOff-ADV, CSII-TRE-Tight-cyclin D1, CSII-TRE-Tight-hCDK4R24C, CSII-CMV-hTERT) (Table 1)

To obtain endodermic cells from iPSCs, three steps were employed (Fig. 1A–C). After iPSCs were differentiated into endodermic cells and EpCAM-positive differentiated cells were sorted by magnetic activation cell sorting, they were infected with lentiviral vectors carrying different combinations of TERT, CDK4R24C, and cyclin D1 genes (Table 1). The infected cells were a mixed population morphologically (Fig. 1D) and were partially positive for CYP3A4 (Fig. 1E). The EpCAM-positive population showed higher levels of E-cadherin expression (Fig. 1F). Among the immortalized cells, iHep4-E8-50 cells exhibited hepatocyte-like morphology, i.e., a polygonal and/or cuboidal shape with tight cell–cell contacts, and were therefore used in subsequent experiments.

**Table 1:** A list of immortalized cells from DILI patients

Cell Name	Gene-1	Gene-2	Gene-3	Gene-4	Parental Cell
iHep2-D9-81	CSII-CMV-hCDK4R24C	CSII-CMV-cyclin D1	CSII-CMV-hTERT		iPSC-I
iHep2-D9-85	CSII-ALB-tetOff-ADV	CSII-TRE-Tight-cyclin D1	CSII-TRE-Tight-hCDK4R24C	CSII-CMV-hTERT	iPSC-I
iHep3-D9-89	CSII-CMV-hCDK4R24C	CSII-CMV-cyclin D1	CSII-CMV-hTERT		iPSC-O
iHep3-E1-29	CSII-ALB-tetOff-ADV	CSII-TRE-Tight-cyclin D1	CSII-TRE-Tight-hCDK4R24C	CSII-CMV-hTERT	iPSC-O
iHep2-E2-64	CSII-CMV-hCDK4R24C	CSII-CMV-cyclin D1	CSII-CMV-hTERT		iPSC-I
iHep2-E2-66	CSII-ALB-tetOff-ADV	CSII-TRE-Tight-hCDK4R24C	CSII-TRE-Tight-cyclin D1	CSII-CMV-hTERT	iPSC-I
iHep3-E2-68	CSII-CMV-hCDK4R24C	CSII-CMV-cyclin D1	CSII-CMV-hTERT		iPSC-O
iHep3-E2-70	CSII-ALB-tetOff-ADV	CSII-TRE-Tight-hCDK4R24C	CSII-TRE-Tight-cyclin D1	CSII-CMV-hTERT	iPSC-O
iHep2-E3-32	CSII-CMV-hCDK4R24C	CSII-CMV-cyclin D1	CSII-CMV-hTERT		iPSC-I
iHep2-E3-34	CSII-ALB-tetOff-ADV	CSII-TRE-Tight-cyclin D1	CSII-TRE-Tight-hCDK4R24C	CSII-CMV-hTERT	iPSC-I
iHep2-E3-36	CSII-CMV-hCDK4R24C	CSII-CMV-cyclin D1	CSII-CMV-hTERT		iPSC-I
iHep3-E3-38	CSII-CMV-hCDK4R24C	CSII-CMV-cyclin D1	CSII-CMV-hTERT		iPSC-O
iHep3-E3-40	CSII-ALB-tetOff-ADV	CSII-TRE-Tight-cyclin D1	CSII-TRE-Tight-hCDK4R24C	CSII-CMV-hTERT	iPSC-O
iHep3-E3-42	CSII-CMV-hCDK4R24C	CSII-CMV-cyclin D1	CSII-CMV-hTERT		iPSC-O
iHep2-E3-62	CSII-ALB-tetOff-ADV	CSII-TRE-Tight-hCDK4R24C	CSII-TRE-Tight-cyclin D1	CSII-CMV-hTERT	iPSC-I
iHep3-E3-64	CSII-ALB-tetOff-ADV	CSII-TRE-Tight-hCDK4R24C	CSII-TRE-Tight-cyclin D1	CSII-CMV-hTERT	iPSC-O
iHep4-E4-72	CSII-CMV-hCDK4R24C	CSII-CMV-cyclin D1	CSII-CMV-hTERT		iPSC-K
iHep4-E4-74	CSII-ALB-tetOff-ADV	CSII-TRE-Tight-cyclin D1	CSII-TRE-Tight-hCDK4R24C	CSII-CMV-hTERT	iPSC-K
iHep4-E4-80	CSII-CMV-hTERT	CSII-CMV-hCDK4R24C	CSII-CMV-cyclin D1		iPSC-K
iHep4-E4-82	CSII-CMV-hTERT	CSII-CMV-hCDK4R24C	CSII-CMV-cyclin D1		iPSC-K
iHep4-E4-84	CSII-ALB-tetOff-ADV	CSII-TRE-Tight-cyclin D1	CSII-TRE-Tight-hCDK4R24C	CSII-CMV-hTERT	iPSC-K
iHep4-E5-50	CSII-CMV-TetOff-ADV	CSII-TRE-Tight-cyclin D1	CSII-TRE-Tight-hCDK4R24C	CSII-CMV-hTERT	iPSC-K
iHep2-E8-45	CSII-ALB-tetOff-ADV	CSII-TRE-Tight-hCDK4R24C	CSII-TRE-Tight-cyclin D1	CSII-CMV-hTERT	iPSC-I
iHep2-E8-46	CSII-ALB-tetOff-ADV	CSII-TRE-Tight-hCDK4R24C	CSII-TRE-Tight-cyclin D1	CSII-CMV-hTERT	iPSC-I
iHep4-E8-49	CSII-CMV-TetOff-ADV	CSII-TRE-Tight-cyclin D1	CSII-TRE-Tight-hCDK4R24C	CSII-CMV-hTERT	iPSC-K
iHep4-E8-50	CSII-CMV-TetOff-ADV	CSII-TRE-Tight-cyclin D1	CSII-TRE-Tight-hCDK4R24C	CSII-CMV-hTERT	iPSC-K

### Puromycin selection of differentiated cells from liver injury-derived iPSC-K

**Fig. 2:**
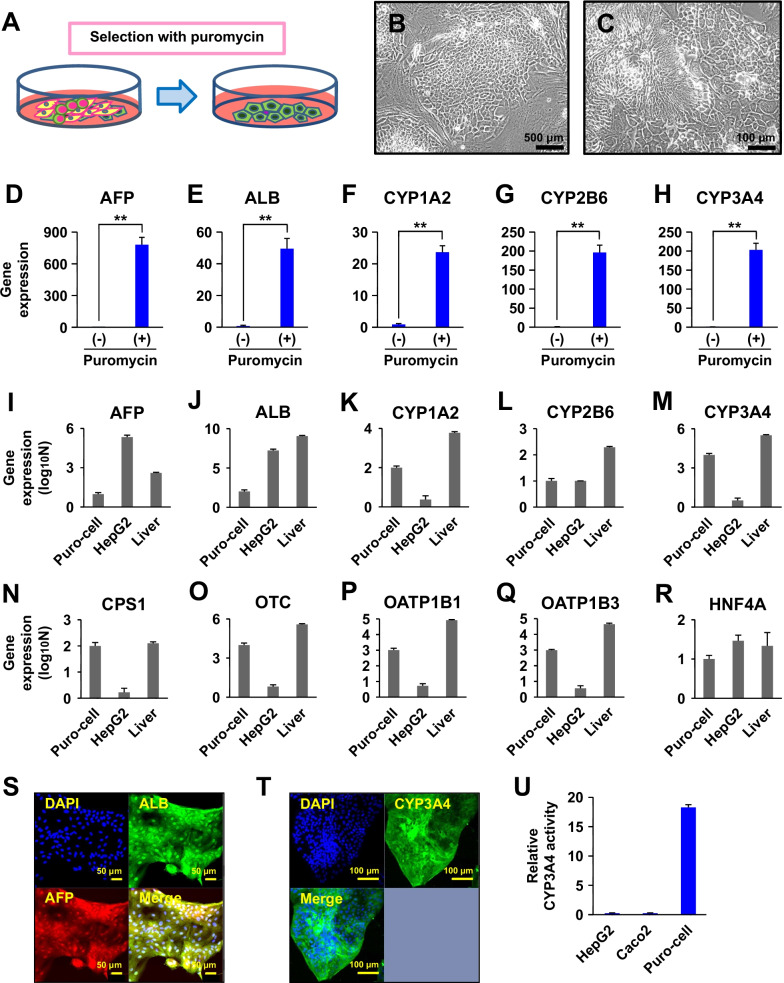
Selection of puromycin-resistant cells. **A** Scheme for selection of puromycin-resistant cells (green) from a mixed population of immortalized cells. For selection, cells were treated with puromycin (3 μg/ml) for 3 days. **B**, **C** Phase-contrast micrograph of puromycin-selected cells. B: Low-power view. C: High-power view. **D**–**H** Quantitative RT-PCR analysis of the genes for AFP (**D**), ALB (E), CYP1A2 (**F**), CYP2B6 (**G**), and CYP3A4 (**H**) after the puromycin treatment. The immortalized differentiated cells (Fig. 1A) were exposed to puromycin at a final concentration of 3 μg/ml for 3 days. RNAs were isolated from the cells 1 week after the puromycin treatment. (−): no puromycin treatment, (+): puromycin treatment. The amounts of each gene at the cells without puromycin treatment were regarded as equal to 1. Statistical analysis was performed using the unpaired two-tailed Student's *t*-test. **P < 0.01. **I**–**R** Quantitative RT-PCR analysis of the puromycin-selected immortalized cells after propagation (Puro-cell), HepG2 cells, and hepatocytes (Liver). **I**: AFP, **J**: ALB, **K**: CYP1A2, **L**: CYP2B6, **M**: CYP3A4, **N**: CPS1, **O**: OTC, **P**: OATP1B1, **Q**: OATP1B3, **R**: HNF4A. **S** Immunocytochemistry of puromycin-selected cells with the antibodies to ALB (green) and AFP (red). **T** Immunocytochemistry of puromycin-selected cells with the antibodies to CYP3A4 (green). **U** CYP3A4 enzymatic activity in HepG2 cells, Caco2 cells, and the puromycin-selected cells

Puromycin is an antibiotic that is toxic to eukaryotic cells and can be metabolized by cytochrome P450. To isolate cells with high expression of CYP3A4 gene from EpCAM positive cells, the cells were exposed to puromycin (3 μg/ml) for 3 days (Fig. 2A). After differentiation of iPSC-K cells, up to 70% of the cells showed hepatocyte-like morphology. Non-hepatocyte-like cells began to die within 1 day after puromycin treatment. On the third day, non-hepatocyte-like cells were essentially undetectable. After 3 days of puromycin treatment and subsequent media exchange, puromycin-resistant cells started to proliferate and generated colonies where the non-hepatocyte-like cells had been. The surviving cells showed morphological characteristics of hepatocytes (Figs. 2B, C). With this selection method, cell populations with parenchymal and epithelial cell morphologies were successfully obtained. The selected cells exhibited endodermal cell morphologies, i.e., normal hepatocytic morphology: clear, distinct one or two round nuclei with prominent nucleoli, dark cytoplasm, and confluent compact monolayer colonies.

### Changes in gene expression by puromycin selection

To determine whether drug selection by puromycin affects gene expression, qRT-PCR analysis was performed. The puromycin-selected cells exhibited higher expression of the hepatocyte-associated genes (Fig. 2D–H). Puromycin is a substrate for CYP3A4, and the expression of CYP3A4 was increased more than 100-fold after puromycin treatment (Fig. 2H). Likewise, the expression of other cytochrome P450s, CYP1A2 and CYP2B6, was increased 10- and 100-fold, respectively (Figs. 2F, G). The expression of the genes for AFP (produced in the embryonic liver) and ALB (produced in the adult liver) was also markedly upregulated (Fig. 2D, E). The ratio of the cells with hepatocyte-like morphology was increased after puromycin treatment. The expression of each gene in puromycin-selected cells was then compared with HepG2 cells and hepatocytes (Fig. 2I–R). The puromycin-selected cells expressed a lower level of the AFP gene than HepG2 cells, and a lower level of the ALB genes than the hepatocyte sample; the puromycin-selected cells expressed comparable levels of the genes for CYP1A2, CYP2B6, CYP3A4, CPS1, OTC, OATP1B1, OATP1B3, and HNF4A, compared with the hepatocyte sample. The puromycin-selected cells were immunocytochemically positive for AFP, ALB, and CYP3A4 (Fig. 2S, T). In addition, the activity of CYP3A4 metabolic enzymes in HepaSM cells was quantitatively analyzed and the results were compared with the activity observed in HepG2 and Caco2 cells (Fig. 2U).

### Characterization of the puromycin-selected cells

**Fig. 3:**
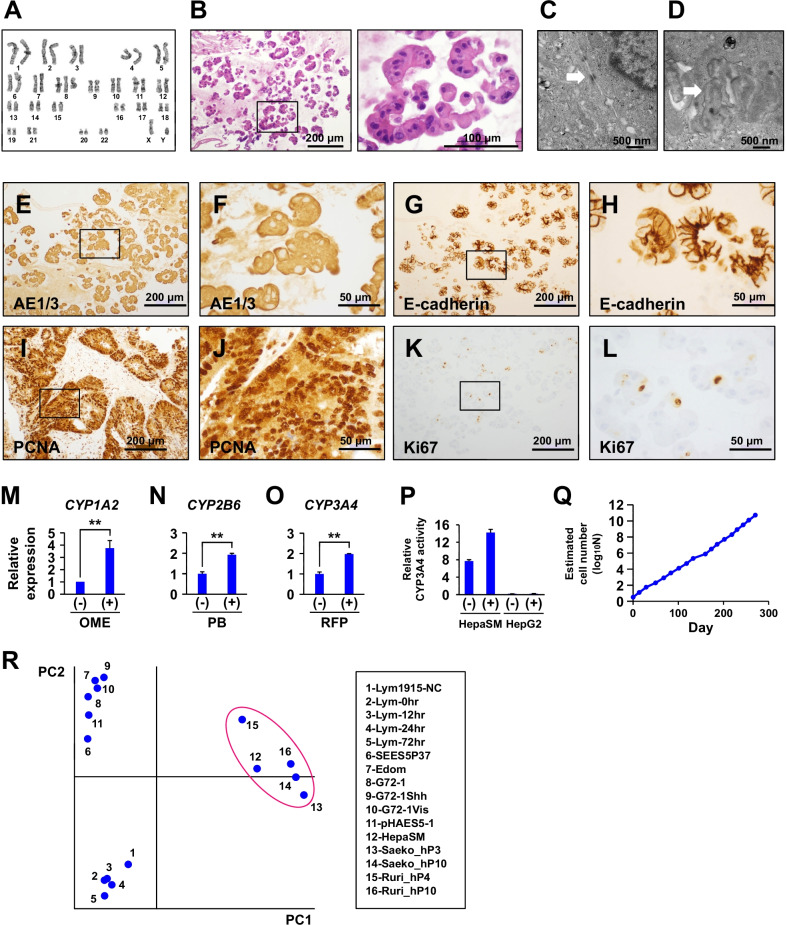
Characterization of puromycin-selected cells. **A** Karyotypic analysis of puromycin-selected cells at passage 10. **B** Histological analysis of puromycin-selected cells embedded in iPGell. The puromycin-selected cells exhibited hepatocyte-like morphology. HE stain. **C**, **D** Ultrastructural analysis. **C**: Desmosome (arrow), **D**: Microvilli (arrow). **E**–**L** Immunocytochemistry of the puromycin-selected cells with the antibodies to AE1/AE3 (**E**, **F**), E-cadherin (**G**, **H**), PCNA (**I**, **J**), and Ki67 (**K**, **L**). Panel **F**, **H**, **J**, and **L** are high-power views of Panel **E**, **G**, **I**, and **K**, respectively. **M** Quantitative RT-PCR analysis of the CYP1A2 gene on the puromycin-selected immortalized cells with exposure to 50 μM omeprazole (OME) for 24 h. mRNA levels were normalized using ubiquitin expression as a housekeeping gene. Statistical analysis was performed using the unpaired two-tailed Student's *t*-test. **P < 0.01. **N** Quantitative RT-PCR analysis of the CYP2B6 gene on the puromycin-selected immortalized cells with exposure to 500 μM phenobarbital (PB) for 48 h. Statistical analysis was performed using the unpaired two-tailed Student's *t*-test. **P < 0.01. **O** Quantitative RT-PCR analysis of the CYP3A4 gene on the puromycin-selected immortalized cells with exposure to 20 μM rifampicin (RFP) for 48 h. Statistical analysis was performed using the unpaired two-tailed Student's *t*-test. **P < 0.01. **P** Induction of CYP3A4 enzymatic activity in the puromycin-selected cells and HepG2 cells exposed to 20 μM rifampicin (RFP) for 48 h. **Q** Growth curve of the puromycin-selected immortalized cells. **R** Principal component analysis of gene expression on lymphangioma-derived cell (1: Lym1915-NC, 2: Lym-0 h, 3: Lym-12 h, 4: Lym-24 h, 5: Lym-72 h), pluripotent stem cells (6: SEES5P37 ESC, 7: Edom iPSCs, 8: G72-1 iPSCs, 9: G72-1Shh iPSC, 10: G72-1Vis iPSC, 11: pHAES5-1 ESC) [26–32], HepaSM cells (12: HepaSM), and hepatocytes (13: Saeko_hP3, 14: Saeko_hP10, 15: Ruri_hP4, 16: Ruri_hP10)

To further characterize the puromycin-selected cells, karyotyping, cytochrome P450 induction test, and long-term proliferation analysis were performed. The karyotypic analysis showed that the puromycin-selected cells had intact chromosomes except for chromosome 8 trisomy in the cell stock (Fig. 3A). The puromycin-selected cells displayed an eosinophilic cytoplasm and round nuclei with dispersed chromatin in a form of gel (Fig. 3B). Ultrastructural analysis revealed desmosomes at the cell junctions and microvilli on the cell surfaces (Fig. 3C, D). However, the cells neither uptake ICG nor accumulate glycogen (Additional file 2: Figure S1). The puromycin-selected cells were positive for CK8/18 (AE1/3) (Fig. 3E, F), E-cadherin (Fig. 3G, H), PCNA (Fig. 3I, J), and Ki67 (Fig. 3K, L). The induction of cytochrome P450 was then assessed in the puromycin-selected cells. The expression levels of three major cytochrome P450 enzymes, CYP1A2, CYP2B6, and CYP3A4, were investigated (Fig. 3M–O). The puromycin-selected cells were exposed to omeprazole for 24 h, phenobarbital for 48 h, and rifampicin for 48 h. Expression of the CYP1A2 gene was upregulated 3.7-fold upon exposure to omeprazole (Fig. 3M); expression of the CYP2B6 gene was upregulated 1.9-fold with exposure to phenobarbital (Fig. 3N); expression of the CYP3A4 gene was upregulated 2.0-fold after exposure to rifampicin (Fig. 3O). The CYP3A4 activity was quantitatively measured in HepaSM and HepG2 cells that had been treated with rifampicin (Fig. 3P). CYP3A4 enzymatic activity was increased approximately 2.0-fold after exposure to rifampicin. In addition to the CYP induction experiments, the potential application of these cells was investigated for hepatotoxic drug testing. The cells were exposed to acetaminophen, trovafloxacin, and levofloxacin for 3 days. No cytotoxicity was observed with any of the drugs used. Long-term cultivation analysis revealed that the puromycin-selected cells continued to proliferate up to at least 36 population doublings for more than 270 days (Fig. 3Q). To clarify the differentiation status of puromycin-selected cells (HepaSM cells), the gene expression levels were compared. PCA demonstrated that HepaSM cells can be classified into the category that includes hepatocytes (circled in red in Fig. 3R). This similarity suggests that HepaSM cells maintain a specific differentiated state that strongly resembles hepatocytes.

## Discussion

Hepatocytes can be accurately evaluated for drug-mediated CYP3A4 induction; this is the gold standard for in vitro hepatic toxicology testing. In this study, we hypothesized that the cells expressing cytochrome P450 genes could survive exposure to cytotoxic antibiotics because of their enhanced drug-metabolizing activity. Puromycin, one of the cytocidal antibiotics, inhibits protein synthesis through the degradation of the 80S ribosome [33], and competitive inhibition of aminoacyl tRNAs [34–36]. Hepatocytes have high metabolic activity of drugs and predictably have resistance to puromycin. The escape of PSC-derived differentiated cells from puromycin is therefore explained by endogenous drug metabolic activity, i.e., expression of the cytochrome P450 genes [37, 38]. To enrich hepatocytes, selection with cell surface markers such as EpCAM by flow cytometric analysis and magnetic cell sorting can also be used [39]. Introduction of cell type-specific expression of cytotoxic antibiotic-resistant genes such as the neomycin-resistant gene is also available for cell selection [40]. Compared with these sophisticated approaches, successful enrichment with puromycin exposure used in this study is simple and straightforward. Indeed, the puromycin selection method can be applied not only to PSC-derived cells, but also to primary hepatocytes and immortalized cell lines [38]. It is also noteworthy that puromycin selection may increase homogeneity or decrease heterogeneity among a lot from the viewpoint of drug metabolism activity.

Upregulation of the CYP3A4 gene, one of the drug-metabolizing genes, after puromycin exposure is convincing, because the cells that are able to metabolize puromycin can be selected. In contrast to the cytochrome P450 genes, increased expression of the albumin gene would not be assumed with puromycin selection. Zone I and III hepatocytes are involved in ammonia metabolism and drug metabolism, respectively, and the ALB gene is considered to be expressed in the zone I hepatocytes [41]. This increase of the ALB gene expression after puromycin-based enrichment of PSC-derived hepatocytes is explained by an increased number of the PSC-derived zone I hepatocytes with an expression of the cytochrome P450 gene. Alternatively, the puromycin-selected zone III hepatocytes start to express the ALB gene. This coordinated change of these genes is aligned with the selection with ammonia, another cytotoxic molecule [37]. In the ammonia-selected cells, not only ammonia metabolizing enzymes are upregulated, but also the expression of cytochrome P450 genes characteristic of zone III hepatocytes is upregulated. During the differentiation of human pluripotent stem cells into hepatocytes, non-hepatocytes are mixed. CYP3A4 enzymatic activity is present in enterocytes of the small intestine and colon, in addition to hepatocytes [42]. It is possible that enterocytes of the small intestine and colon are selected by puromycin along with hepatocytes. The absence of enterocyte marker gene expression suggests that the immortalized cells (HepaSM cells) are hepatocytes, while some of the immortalized line cells have enterocytic features, which will be the subject of further analysis.

The immortalized cells were introduced with three genes, i.e., human cyclin D1, mutant CDK4, and telomerase, which is another successful example of the establishment of useful cells with stable proliferation and genetic stability [15, 16]. Oncogenic genes such as simian vacuolating virus 40 large T antigen, polyomavirus middle T antigen, or human TERT with human papillomavirus type 16-derived E6/E7 proteins can immortalize cells, but they may reduce genetic stability, and the original cell characteristics may be lost or altered [43]. Human hepatocytes with their specific function are maintained in vitro for an extended period of time, using small-molecule combinations [44]. Likewise, a culture system by a defined medium is established to proliferate human hepatocytes with functionality and engraftability continually for more than one month with a 10,000-fold expansion in numbers [45]. Human hepatic progenitor cells can be obtained from human infant hepatocytes using the strategy with a cocktail of small-molecule signaling inhibitors [46]. Similarly, functional and transplantable hepatocytes can be generated from human endoderm stem cells or embryonic stem cells, using a serum-free suspension differentiation system that enables efficient large-scale generation [47]. Thus, it is necessary to investigate whether puromycin-selected hepatocytes can also be proliferated by setting appropriate culture conditions or treating them with small-molecule compounds. Puromycin-selected cells with drug-mediated CYP3A4 induction potency from a patient with DILI may serve as a model cell for multi-physiological systems, an organ/organoid on a chip, or body on a chip for examination of drug metabolism and toxicity [48].

## Conclusions

From the viewpoint of the extremely high CYP3A4 level, the puromycin-selected immortalized cells are ideal model cells for in vitro drug toxicology testing. Because CYP3A4 contributes to the first-pass metabolism of many commercial drugs, it is important to investigate the CYP3A4-mediated metabolism to estimate hepatotoxicity. It is known that the CYP3A4 expression can be induced by various drugs, such as dexamethasone, PB, RFP, and 1α,25-dihydroxyvitamin D3. The induction of CYP3A4 expression by such drugs might affect the pharmacokinetics of concomitant drugs administered orally. Our cell model with reproducible induction of CYP3A4 could, therefore, be useful for an early stage of drug development. Another advantage of this cell model is that it does not change during in vitro propagation and is thus readily available for cytochrome P450 induction tests and cell toxicity examination. Finally, this robust system for toxicology testing should yield a better cost-performance balance.

## Supplementary Information

**Additional file 1: Table S1.** Primer pairs and experimental conditions for RT-PCR.

**Additional file 2. Figure S1.** Indocyanine green (ICG) uptake test and periodic acid-Schiff (PAS) staining of iPSC-derived cells (SM). **A**-**H**. ICG uptake test of control hepatocytes (A-D: Hep2007) and iPSC-derived cells (E–H: SM). The cells were washed with PBS and incubated in DMEM medium containing 1 mg/ml freshly prepared ICG reagent at 37 °C for 1 h. At least 10 non-overlapping fields of view were recorded under the microscope and cells with green nuclear staining were counted as positive. Cells were then incubated with ICG-free complete medium at 37 °C for 6 h to detect ICG release. A, B, E, F: Phase-contrast micrograph. C, D, G, H: Fluorescent micrographs. A, C, E, G: Low-power views. B, D, F, H: High-power views. **I**. PAS stain of iPSC-derived cells (SM). Cells were fixed with 4% paraformaldehyde for 10 min. Following washing with PBS, cells were incubated with 0.5% periodic acid solution for 5 min, then stained with Schiff's reagent for 15 min, followed by counterstaining with hematoxylin solution for 2 min. The cytoplasm of the puromycin-treated cells did not stain purple-red.

## Data Availability

The datasets used and/or analyzed during the current study are available from the corresponding author on reasonable request.
