# Supplementary material for: Puromycin-based purification of cells with high expression of the cytochrome P450 CYP3A4 gene from a patient with drug-induced liver injury (DILI)
Source: Stem Cell Res Ther. 2022 Jan 10;13:6. doi: 10.1186/s13287-021-02680-4 (PMC8744258; doi:10.1186/s13287-021-02680-4)
Supplement: Supplementary file 1 — Additional file 1: Table S1. Primer pairs and experimental conditions for RT-PCR. [file 13287_2021_2680_MOESM1_ESM.docx]

# Table S1. Primer pairs and experimental conditions for RT-PCR

| Gene product | Forward and reverse primers (5'–3') | Expected product size (bp) | |
| --- | --- | --- | --- |
| AFP | TGCAGCCAAAGTGAAGAGGGAAGA  CATAGCGAGCAGCCCAAAGAAGAA | 217 |  |
| ALB | TGCTTGAATGTGCTGATGACAGGG  AAGGCAAGTCAGCAGGCATCTCATC | 162 |  |
| CYP1A2 | CAATCAGGTGGTGGTGTCAG  GCTCCTGGACTGTTTTCTG | 245 |  |
| CYP2B6 | TCCTTTCTGAGGTTCCGAGA  TCCCGAAGTCCCTCATAGTG | 416 |  |
| CYP3A4 | CAAGACCCCTTTGTGGAAAA  CGAGGCGACTTTCTTTCATC | 187 |  |
| OTC | TTTCCAAGGTTACCAGGTTACAA  CTGGGCAAGCAGTGTAAAAAT | 78 |  |
| CPS1 | CAAGTTTTGCAGTGGAATCG  GGACAGATGCCTGAGCCTAA | 115 |  |
| HNF4α | CATGGCCAAGATTGACAACCT  TTCCCATATGTTCCTGCATCAG | 113 |  |
| UBIQUITIN | GGAGCCGAGTGACACCATTG  CAGGGTACGACCATCTTCCAG | 118 |  |
| OATP1B1 | GAATGCCCAAGAGATGATGCTT  AACCCAGTGCAAGTGATTTCAAT | 154 |  |
| OATP1B3 | GTCCAGTCATTGGCTTTGCA  CAACCCAACGAGAGTCCTTAGG | 111 |  |
