# Supplementary figures and images for: Puromycin-based purification of cells with high expression of the cytochrome P450 CYP3A4 gene from a patient with drug-induced liver injury (DILI)
Source: Stem Cell Res Ther. 2022 Jan 10;13:6. doi: 10.1186/s13287-021-02680-4 (PMC8744258; doi:10.1186/s13287-021-02680-4)

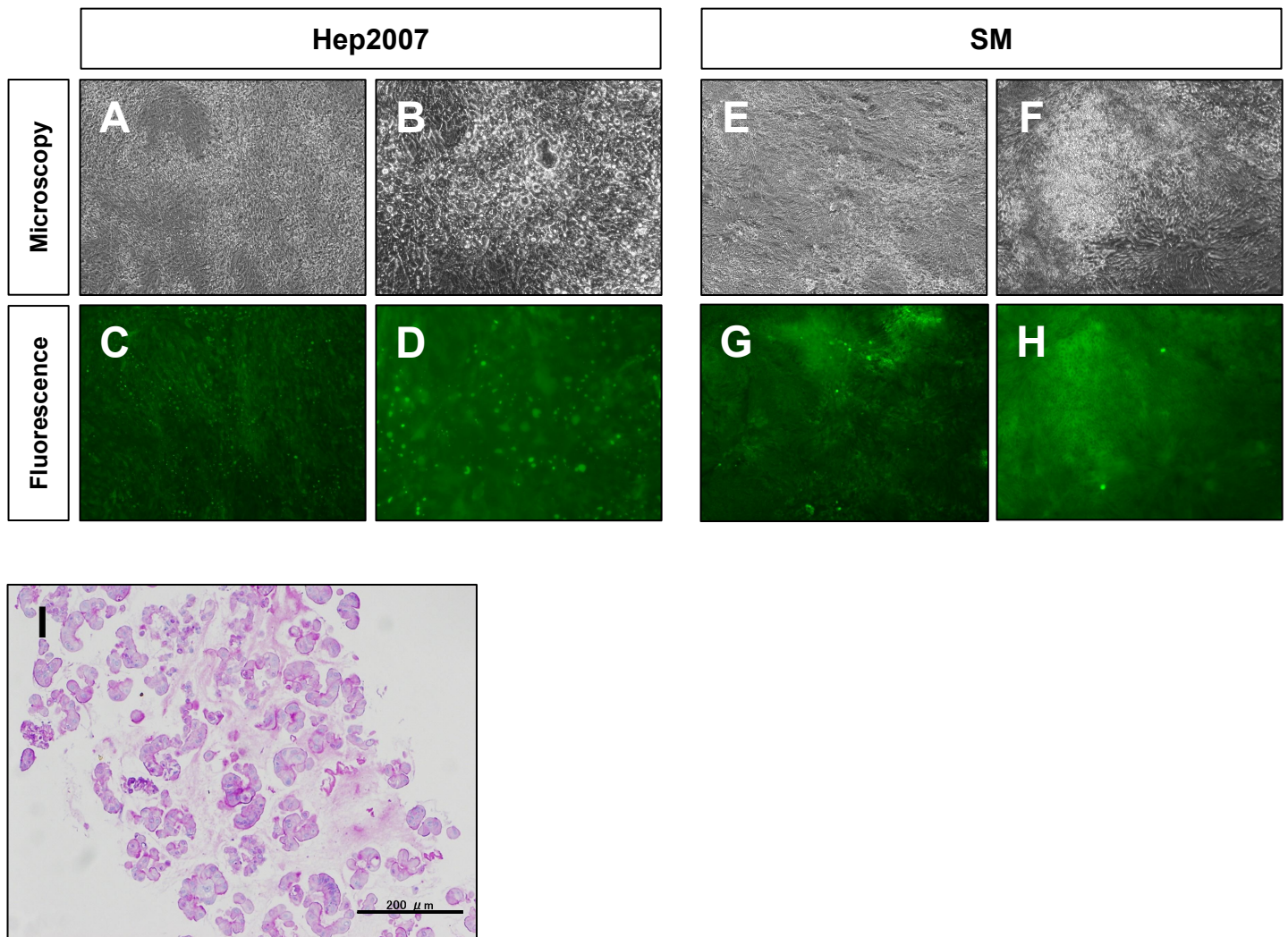

**Figure S1**

Supplement: Supplementary file 2 — Additional file 2. Figure S1. Indocyanine green (ICG) uptake test and periodic acid-Schiff (PAS) staining of iPSC-derived cells (SM). A-H. ICG uptake test of control hepatocytes (A-D: Hep2007) and iPSC-derived cells (E–H: SM). The cells were washed with PBS and incubated in DMEM medium containing 1 mg/ml freshly prepared ICG reagent at 37 °C for 1 h. At least 10 non-overlapping fields of view were recorded under the microscope and cells with green nuclear staining were counted as positive. Cells were then incubated with ICG-free complete medium at 37 °C for 6 h to detect ICG release. A, B, E, F: Phase-contrast micrograph. C, D, G, H: Fluorescent micrographs. A, C, E, G: Low-power views. B, D, F, H: High-power views. I. PAS stain of iPSC-derived cells (SM). Cells were fixed with 4% paraformaldehyde for 10 min. Following washing with PBS, cells were incubated with 0.5% periodic acid solution for 5 min, then stained with Schiff's reagent for 15 min, followed by counterstaining with hematoxylin solution for 2 min. The cytoplasm of the puromycin-treated cells did not stain purple-red. [file 13287_2021_2680_MOESM2_ESM.pdf]
